# Highly Selective Detection of Benzene and Discrimination of Volatile Aromatic Compounds Using Oxide Chemiresistors with Tunable Rh‐TiO_2_ Catalytic Overlayers

**DOI:** 10.1002/advs.202004078

**Published:** 2021-02-01

**Authors:** Young Kook Moon, Seong‐Yong Jeong, Young‐Moo Jo, Yong Kun Jo, Yun Chan Kang, Jong‐Heun Lee

**Affiliations:** ^1^ Department of Materials Science and Engineering Korea University Seoul 02841 Republic of Korea

**Keywords:** benzene, bilayer sensor, gas sensors, Rh‐TiO_2_, volatile aromatic compounds

## Abstract

Volatile aromatic compounds are major air pollutants, and their health impacts should be assessed accurately based on the concentration and composition of gas mixtures. Herein, novel bilayer sensors consisting of a SnO_2_ sensing layer and three different *x*Rh‐TiO_2_ catalytic overlayers (*x* = 0.5, 1, and 2 wt%) are designed for the new functionalities such as the selective detection, discrimination, and analysis of benzene, toluene, and *p*‐xylene. The 2Rh‐TiO_2_/SnO_2_ bilayer sensor shows a high selectivity and response toward ppm‐ and sub‐ppm‐levels of benzene over a wide range of sensing temperatures (325–425 °C). An array of 0.5Rh‐, 1Rh‐, and 2Rh‐TiO_2_/SnO_2_ sensors exhibits discrimination and composition analyses of aromatic compounds. The conversion of gases into more active species at moderate catalytic activation and the complete oxidation of gases into non‐reactive forms by excessive catalytic promotion are proposed as the reasons behind the enhancement and suppression of analyte gases, respectively. Analysis using proton transfer reaction‐quadrupole mass spectrometer (PTR‐QMS) is performed to verify the above proposals. Although the sensing characteristics exhibit mild moisture interference, bilayer sensors with systematic and tailored control of gas selectivity and response provide new pathways for monitoring aromatic air pollutants and evaluating their health impacts.

## Graphical abstract

Bilayer sensors with tunable Rh‐TiO_2_ catalytic overlayers have been proposed for highly selective detection of benzene and discrimination of volatile aromatic compounds. The sensors and sensors array in this study will open up a new avenue for accurate indoor air quality monitoring, gas leak detection in the petroleum industry, and assessment of air pollution in congested areas and gas stations.

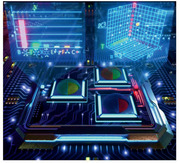

## 1 Introduction

Oxide semiconductor chemiresistors with the irreplaceable advantages of high, rapid, and reversible response, along with simple structure, cost‐effectiveness, and easy miniaturization have been widely used to detect various toxic, explosive, and environmental gases.^[^ ^1^, ^2^, ^3^, ^4^, ^5^, ^6^, ^7^, ^8^, ^9^, ^10^ ^]^ However, a simple gas‐sensing algorithm involving charge transfer between an oxide surface and analyte gas often impedes the selective detection of a specific gas. The selectivity issue becomes significant when one wants to detect a stable gas such as benzene with low reactivity. To overcome this limitation, an electronic nose—an array of sensors with partial gas selectivity—has been suggested to distinguish between different gases or odors.^[^ ^11^, ^12^, ^13^, ^14^, ^15^ ^]^ However, most electronic noses do not quantify gas concentrations.^[^ ^16^ ^]^ Thus, the discriminative quantification of gases using oxide chemiresistors has been a long‐standing problem.

**Figure 1:**
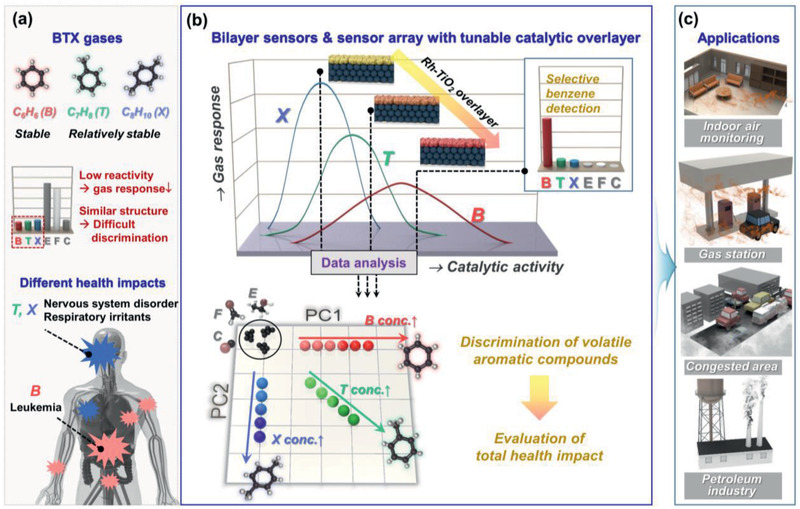
Concept of study.

The quantitative and discriminative detection of representative aromatic compounds such as benzene, toluene, and xylene (BTX) gases using chemiresistors is a challenge for monitoring air quality.^[^ ^17^, ^18^ ^]^ In indoor environments, the responses of oxide chemiresistors to BTX gases with relatively low reactivity are generally lower than those to interfering formaldehyde (F) and ubiquitous ethanol (E) (**Figure** **1**a, upper), both of which have high reactivity.^[^ ^19^, ^20^ ^]^ Furthermore, the similar chemical structures of benzene ring‐containing BTX molecules often impede their distinction in indoor and outdoor atmospheres.^[^ ^21^, ^22^ ^]^ The various health impacts of BTX gases hamper the accurate evaluation of the total health impacts from these aromatic pollutants (Figure 1a, lower).^[^ ^23^, ^24^ ^]^ Benzene is a carcinogen that induces leukemia, whereas its methyl derivatives are less toxic but still harmful enough to affect the central nervous system, eyes, and respiratory system. Thus, the exposure limits of BTX gases vary widely. Unfortunately, most oxide‐based gas sensors exhibit the lowest response to the most toxic benzene because of its extremely high stability. Therefore, the discriminative quantification of BTX gases is necessary for accurate indoor air quality monitoring as well as diverse outdoor applications, such as leak detection in the petroleum industry, monitoring of air pollution in congested areas, and assessment of air quality at gas stations.

In a previous work, we reported bilayer sensors consisting of oxide semiconductor sensing film and catalytic overlayers as platforms to modulate the gas response and selectivity,^[^ ^25^, ^26^, ^27^, ^28^ ^]^ and suggested the catalytic reforming and oxidation of analyte gases as the reasons behind the enhancement and suppression of the gas response, respectively. However, the reforming and oxidation of analyte gases have not been investigated experimentally because of difficulties in the real‐time analysis of ppm‐level gas and the electronic nose using the array of bilayer sensors with tunable catalytic overlayers has never been designed. Herein, we report that bilayer sensors consisting of a SnO_2_ thick film with Rh‐TiO_2_ catalytic overlayers (as well as an array constructed by these sensors) exhibit three new capabilities: highly selective detection of carcinogenic benzene, discrimination of BTX gases, and compositional analysis of binary mixture of BTX gases under defined environment (Figure 1b). This combination enables precise indoor air monitoring, taking into account the health impacts as well as the tailored monitoring of outdoor BTX gases in petroleum‐associated locations (Figure 1c). The possible reforming and oxidation reactions of ppm‐level aromatic gases at Rh‐TiO_2_ catalytic overlayers with different Rh loading were investigated using proton transfer reaction mass spectrometry (PTR‐MS). The main focuses of this study are directed at the in‐depth understanding of the gas‐sensing mechanisms of bilayer‐design sensors enabling systematic modulation of the response and selectivity to analyte gases, highly selective benzene detection, and discrimination of aromatic compounds.

## 2 Results and Discussion

**Figure 2:**
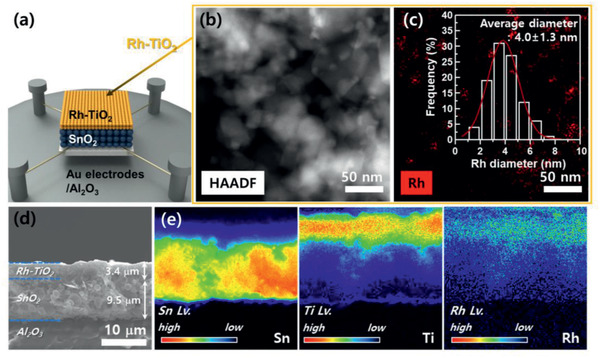
a) Schematic diagram of 2Rh‐TiO_2_/SnO_2_ sensor, b) HAADF–STEM image, and c) elemental mapping of 2Rh‐TiO_2_ catalytic overlayer (inset: size distribution of Rh nanoparticles). d) Cross‐sectional SEM image and e) EPMA elemental (Sn, Ti, and Rh) mapping images of the sensing film.

A schematic of the *x*Rh‐TiO_2_/SnO_2_ (*x* = 0.5–2 wt%) sensor is shown in **Figure** **2**a. The SnO_2_ sensing layer was screen printed on an Al_2_O_3_ substrate with Au electrodes. After drying the SnO_2_ sensing film, the *x*Rh‐TiO_2_ overlayers were coated onto the SnO_2_ film. SnO_2_ hollow spheres were prepared by ultrasonic spray pyrolysis and sequent annealing at 600 °C. The average diameter of ≈100 spheres was 1.35 ± 0.56 µm (Figure S1a, Supporting Information). The brighter contour at the central regions of the spheres than at the outer region in transmission electron microscopy (TEM) image (Figure S1b, Supporting Information) indicates the hollow morphology of the spheres (shell thickness: 18.3 ± 2.9 nm) (inset in Figure S1b, Supporting Information). The tetragonal structure of the SnO_2_ phase was confirmed by lattice fringes exhibiting ($\bar{1}01$) and ($\bar{1}10$) planes with interplanar spacings of 2.7 and 3.6 Å (Figure S1c, Supporting Information) and the X‐ray diffraction (XRD) pattern (Figure S1d, Supporting Information). The Brunauer–Emmett–Teller (BET) specific surface area of the SnO_2_ hollow spheres was 10.01 m^2^ g^−1^ and the N_2_ adsorption and desorption isotherms were type IV with H3 hysteresis loops, demonstrating the high gas accessibility of SnO_2_ hollow spheres with abundant mesopores (Figure S1e, Supporting Information).

Rh‐TiO_2_ powders were prepared by impregnation of a Rh source on the TiO_2_ powders and subsequent heat treatment at 500 °C for 2 h. The Rh contents of the 0.5Rh‐TiO_2_, 1Rh‐TiO_2_, and 2Rh‐TiO_2_ samples measured by inductively coupled plasma optical emission spectrometry were 0.43, 0.92, and 1.83 wt%, respectively. XRD analysis revealed that all three samples after heat treatment were a mixture of rutile (ICDD # 21–1276) and anatase (ICDD # 21–1272) phases (Figure S2, Supporting Information). No Rh‐associated peak was found even in the 2Rh‐TiO_2_ sample, which can be attributed to either the low detection limit of XRD or highly dispersed Rh nanoparticles. However, the uniform dispersion of Rh nanoparticles could be clearly observed in high‐angle annular dark‐field scanning transmission electron microscopy (HAADF–STEM) and mapping images of the 2Rh‐TiO_2_ sample (particles with brighter contour in Figure 2b and red dots in Figure 2c). The average size of the Rh nanoparticles was 4.0 ± 1.3 nm (Figure 2c inset). High‐resolution TEM images reconfirmed that the Rh nanoparticles with high crystallinity were loaded onto TiO_2_ (Figure S3a,b, Supporting Information). To verify the chemical state of the 2Rh‐TiO_2_ sample, the sample annealed at 500 °C for 2 h was analyzed using X‐ray photoelectron spectroscopy. The Ti 2p_1/2_ and Ti 2p_3/2_ peaks at 463.87 and 458.12 eV indicate that the surface of TiO_2_ is mainly in the +4 oxidation state and contains a small portion of Ti^3+^ (Figure S3c, Supporting Information).^[^ ^29^ ^]^ The Rh 3d_3/2_ and 3d_5/2_ peaks at 313.72 and 308.85 eV suggest that most Rh components are oxidized to Rh_2_O_3_ during heat treatment (Figure S3d, Supporting Information).^[^ ^30^ ^]^

The cross‐sectional scanning electron microscopy (SEM) image of the bilayer sensor shows the lower SnO_2_ sensing film (thickness: 9.5 µm) and the catalytic 2Rh‐TiO_2_ overlayer (thickness: 3.4 µm) (Figure 2d). The thicknesses of the SnO_2_ sensing layer and *x*Rh‐TiO_2_ overlayers in all the sensors were similar (Figure 2d; Figure S4, Supporting Information). The electron probe microanalysis (EPMA) element mapping results show that the Sn component is uniformly distributed throughout the SnO_2_ sensing film, whereas the Rh and Ti components are located only in the overlayer (Figure 2e).

The gas responses of pure SnO_2_ and *x*Rh‐TiO_2_/SnO_2_ sensors to 5 ppm of aromatic BTX compounds, and other representative indoor pollutants (ethanol, carbon monoxide (CO), and formaldehyde (HCHO)) were measured at 325–425 °C. All the sensors showed typical gas‐sensing characteristics of n‐type oxide semiconductors; the sensor resistance decreased upon exposure to reducing gases and returned to the baseline when the atmosphere was switched to air (Figure S5, Supporting Information). Accordingly, the gas response (*S*) was defined as (*R*_a_−*R*_g_)/*R*_g_ = (*R*_a_/*R*_g_)−1 (*R*_a_: resistance in air, *R*_g_: resistance in analyte gas).

**Figure 3:**
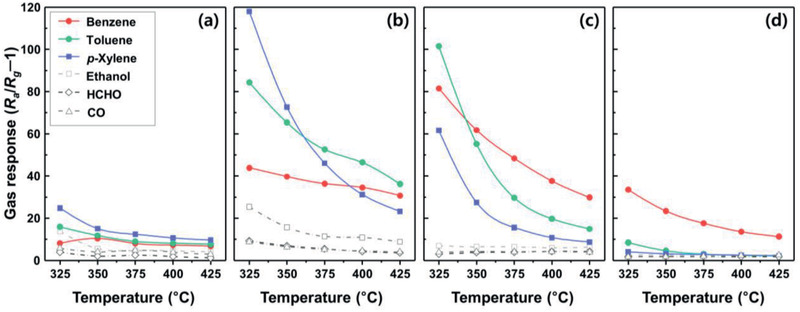
Gas‐sensing characteristics of a) pure SnO_2_, b) 0.5Rh‐TiO_2_/SnO_2_, c) 1Rh‐TiO_2_/SnO_2_, and d) 2Rh‐TiO_2_/SnO_2_ sensors to 5 ppm of various gases (benzene, toluene, *p*‐xylene, ethanol, HCHO, and CO) in the range 325–425 °C.

The gas responses of the pure SnO_2_ sensor to aromatic BTX compounds were slightly higher or similar to those to other gases (ethanol, HCHO, and CO) over a wide range of operating temperatures (325–425 °C) (**Figure** **3**a). In contrast, the gas response of the 0.5Rh‐TiO_2_/SnO_2_ sensor becomes significantly high (Figure 3b). It is worth noting that a higher enhancement was found in the response to aromatic compounds than in that to ethanol, HCHO, and CO. At a low sensing temperature of 325 °C, the responses to aromatic compounds have the order: (*S*_X_ = 117) > (*S*_T_ = 84) > (*S*_B_ = 43) (*S*_X_, *S*_T_, and *S*_B_ are the responses to *p*‐xylene, toluene, and benzene, respectively). With an increase in sensing temperature, however, *S*_X_ decreased sharply, but the decrease in *S*_B_ was gradual, thereby leading to a change in the response order (*S*_T_ > *S*_B_ > *S*_X_) at 425 °C. This demonstrates the potential to tailor the selectivity toward aromatic compounds by modulating the catalytic overlayer and sensing temperature. For the 1Rh‐TiO_2_/SnO_2_ sensor, the response to aromatic compounds remained high, but those of other interference gases decreased further (Figure 3c). It is interesting that compared to the 0.5Rh‐TiO_2_/SnO_2_ sensor, the responses of the 1Rh‐TiO_2_/SnO_2_ sensor to toluene and benzene increased to 101.5 and 81.4, whereas the response to *p*‐xylene decreased to 61.6 at 325 °C. The 2Rh‐TiO_2_/SnO_2_ sensor showed the highest response to benzene, and the response to all other gases decreased to a negligible level (Figure 3d). This suggests that a highly selective detection of benzene over a wide range of sensing temperatures is possible using the 2Rh‐TiO_2_/SnO_2_ sensor.

In the present bilayer sensor configuration, the analyte gas should be transported from the surface by way of a Rh‐TiO_2_ catalytic overlayer to the lower part of SnO_2_ sensing film close to the electrodes for the gas‐sensing reaction. Accordingly, the variation in the gas response with different catalytic overlayers can be understood within the framework of gas transport, the reforming of the analyte gas into more active forms, and/or the complete oxidation of the analyte gas into non‐reactive or less‐reactive species (CO_2_ or H_2_O). In general, the introduction of an overlayer tends to diminish gas transport. Nevertheless, at 325 °C, the response to all aromatic compounds significantly increased with the coating of the 0.5Rh‐TiO_2_ overlayer (Figure 3a,b), suggesting that the gas reforming effect due to moderate catalytic activation is predominant. This is in line with the results of our previous studies on bilayer sensors with catalytic overlayers.^[^ ^26^, ^27^, ^28^ ^]^ Furthermore, considering the order of gas reactivity (X > T > B), the order of gas response (*S*_X_ > *S*_T_ > *S*_B_) in the 0.5Rh‐TiO_2_ overlayer at 325 °C can be explained by the more reforming of gas with higher reactivity. In contrast, the response to all other gases except highly stable benzene decreased to a negligible level in the 2Rh‐TiO_2_/SnO_2_ sensor (Figure 3d), which can be attributed to the oxidation of gases into non‐reactive products due to the excessive loading of the catalysts. The 90% response and 90% recovery times (*τ*_res_ and *τ*_recov_), the times to reach 90% of resistance variation upon exposure to analyte gas and air, were calculated from the sensing transients at 325, 375, and 425 °C (Figure S6, Supporting Information). In pristine SnO_2_ sensor, the *τ*_res_ values ranged from 4 to 12 s and the *τ*_recov_ values ranged from 73 to 873 s. The *τ*_res_ and *τ*_recov_ values of SnO_2_ sensor with xRh‐TiO_2_ overlayers were comparable or slightly higher.

To analyze and understand the gas‐sensing reaction, the responses to aromatic compounds were plotted as a function of catalyst loading (Figure S7, Supporting Information). With an increase in the amount of catalytic Rh in the overlayer, the responses to the aromatic compounds tend to increase, reach a maximum, and then decrease, supporting the above explanation on gas reforming and oxidative filtering. In the bell‐shaped curves, the amount of Rh loading on TiO_2_ to show the maximum gas response (Rh_M_) is associated with the reactivity of the analyte gas; Rh_M_ for high‐reactivity gas (i.e., *p*‐xylene) is low and Rh_M_ for low‐reactivity gas (i.e., benzene) is high. Indeed, all the Rh_M_ values in Figure S7, Supporting Information show the order B ≥ T ≥ X. This supports the above explanation. Thus, the highest *p*‐xylene response by 0.5Rh‐TiO_2_/SnO_2_ at 325 °C can be explained by effective gas reforming and the high selectivity toward benzene by 2Rh‐TiO_2_/SnO_2_ at 325 °C can be attributed to the oxidative filtering of other interference gases with higher reactivity.

To investigate the catalytic roles of Rh and TiO_2_ in detail, the gas sensing characteristics of bilayer sensors loaded with different noble metals (0.5Pd‐TiO_2_/SnO_2_, 0.5Pt‐TiO_2_/SnO_2_, and 0.5Au‐TiO_2_/SnO_2_) at 325 °C have been measured and compared with those of 0.5Rh‐TiO_2_/SnO_2_ sensor (Figure S8, Supporting Information). Overall, the coating of 0.5Pd‐TiO_2_ overlayer decreased gas responses probably due to the catalytic oxidation of gases. The 0.5Pt‐TiO_2_ overlayer slightly increased the benzene response but decreased xylene response, indicating the mixed effect of gas reforming and oxidation. Last, the 0.5Au‐TiO_2_ overlayer enhanced BTX responses but the amount of response enhancement was substantially lower than that by 0.5Rh‐TiO_2_ overlayer. The gas reforming of less reactive gases into more reactive forms plays the key role in maximizing the variation of gas response. In this perspective, the Rh‐TiO_2_ catalytic overlayer was the most effective. However, the dissimilar gas sensing behaviors of the sensors with different catalytic overlayers suggest the possibility to control gas sensing patterns.

In order to examine the effect of supporting oxide in catalytic overlayer on gas sensing reaction, the gas‐sensing characteristics of the TiO_2_/SnO_2_ and 2Rh‐Al_2_O_3_/SnO_2_ sensors were investigated in the range 325–425 °C (Figure S9, Supporting Information). The gas response to aromatic compounds slightly increased with the coating of the TiO_2_ overlayer on the SnO_2_ sensing film (Figure 3a; Figure S9a, Supporting Information), which can be attributed to the moderate gas reforming effect by the catalytic TiO_2_. However, the mild gas reforming induced by the TiO_2_ overlayer was insufficient to achieve a high response and selectivity to BTX gases. In comparison, the response to aromatic compounds significantly increased by introducing 2Rh‐Al_2_O_3_ on the SnO_2_ sensing films (Figure S9b, Supporting Information). This supports the idea that the introduction of a Rh‐containing catalytic overlayer is more effective in enhancing the gas response of oxide chemiresistors toward aromatic compounds by reforming less‐reactive gases into more reactive species. However, unlike the 2Rh‐TiO_2_ overlayer (Figure 3d), a 2Rh‐Al_2_O_3_ overlayer did not lead to the complete oxidation of *p*‐xylene and toluene (Figure S9b, Supporting Information), indicating that the catalytic activity of 2Rh‐Al_2_O_3_ is lower than that of 2Rh‐TiO_2_. If the same materials are used, the material with higher surface area would be more advantageous to enhance catalytic activation.^[^ ^31^ ^]^ However, in the present study, the higher catalytic activity was achieved in 2Rh‐TiO_2_ with the lower surface area (Table S1, Supporting Information), suggesting the synergistic catalytic effect of Rh and supporting TiO_2_. Indeed, the catalytic activity of Rh nanoparticles is known to be closely dependent on the oxide support materials.^[^ ^32^, ^33^, ^34^ ^]^ Guan et al.^[^ ^34^ ^]^ reported that the activation energy for the CO oxidation of Rh‐TiO_2_ is significantly lower than those of other Rh‐supported metal oxides such as Al_2_O_3_, SiO_2_, and CeO_2_. This implies that TiO_2_‐supported Rh nanoparticles exhibit superior catalytic activity for the oxidation of reducing gases compared to other Rh‐based catalysts. Furthermore, TiO_2_‐supported noble metal nanoparticles have been widely used for the catalytic oxidation of aromatic compounds because of their high catalytic activity.^[^ ^35^, ^36^ ^]^ Therefore, the TiO_2_ supports and Rh nanoparticles significantly contribute to catalytic promotion, and high benzene selectivity in the 2Rh‐TiO_2_/SnO_2_ sensor can be attributed to the effective oxidation of reactive interference gases using the synergistically combined Rh‐TiO_2_ composite catalysts.

**Figure 4:**
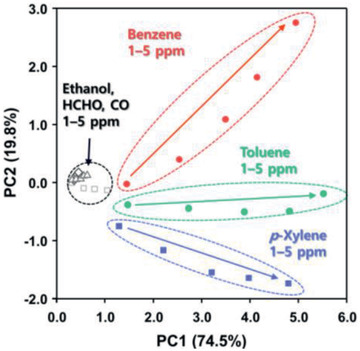
PCA plot using the data from 0.5Rh‐TiO_2_/SnO_2_, 1Rh‐TiO_2_/SnO_2_, and 2Rh‐TiO_2_/SnO_2_ sensors to demonstrate the discrimination of aromatic BTX compounds over the interferences from ethanol, HCHO, and CO (concentration: 1–5 ppm).

It is worth noting that the 0.5Rh‐TiO_2_/SnO_2_ and 1Rh‐TiO_2_/SnO_2_ sensors show different partial selectivity to *p*‐xylene and toluene, whereas the 2Rh‐TiO_2_/SnO_2_ sensor shows high selectivity to benzene. These distinct variations in the gas response with strong tendency can be used for discriminating different aromatic compounds. Principal component analysis (PCA) was performed to identify the aromatic compounds using the gas response of the three xRh‐TiO_2_/SnO_2_ sensors at 325 °C (**Figure** **4**, Figure S10, Supporting Information). The 1–5 ppm of benzene, toluene, and *p*‐xylene were clearly separated with 1–5 ppm of other indoor pollutant gases (ethanol, HCHO, and CO), demonstrating that aromatic compounds could be discriminated. Interestingly, PC1 increased with increasing concentrations of each aromatic compound (benzene, toluene, and *p*‐xylene), successfully demonstrating that not only discrimination but also quantification of aromatic compounds is possible. Furthermore, 18 different compositions of binary mixed gases (B+T, B+X, or T+X) were also analyzed using the PCA method (Figure S11, Supporting Information). All three PCA plots using the gas sensing data for six different compositions of binary gas mixtures (1–6 in Figure S11b, Supporting Information) show the triangular shape, which are similar to three triangles built by the compositions of gas mixtures in Figure S11a, Supporting Information. Furthermore, the relative directions of PCA data from the center of triangles in Figure S11b, Supporting Information, are identical to those of compositional triangles in Figure S11a, Supporting Information, enabling the estimation of binary gas composition by analyzing the PCA data in a well‐defined environment. These results are remarkable considering that the response data are acquired by only three bilayer sensors with different amounts of Rh catalyst in the overlayer.

The distinctive features of the aforementioned sensors and sensor array can shed light on various applications. First, benzene, which is extremely toxic and stable, can be detected in a highly selective and sensitive manner using a single 2Rh‐TiO_2_/SnO_2_ sensor. As mentioned in the introduction, most oxide semiconductor gas sensors exhibit the lowest response to the most hazardous benzene, relatively low responses to harmful toluene and xylene, and high responses to HCHO and ethanol. According to the US Occupational Safety and Health Administration, the permissible exposure limit for the 8 h time‐weighted average for benzene is 1 ppm, whereas those for toluene and xylene are 200 and 100 ppm, respectively.^[^ ^37^, ^38^, ^39^ ^]^ This suggests that the selective detection of benzene is not only challenging but also important. Considering the above, the 2Rh‐TiO_2_/SnO_2_ sensor is an excellent platform for the selective and quantitative detection of highly toxic benzene in the presence of various interference gases such as toluene, xylene, ethanol, HCHO, and CO in indoor environments.

Second, the sensor array enables the analysis of binary mixture of BTX gases. Most sensor arrays for electronic noses are designed to discriminate gas species,^[^ ^40^, ^41^ ^]^ but it is difficult to achieve both the discrimination of gases and quantification of gas concentration.^[^ ^16^ ^]^ If one knows the pollutant species and their concentrations in an indoor atmosphere, the total health impact from all harmful pollutants can be predicted. For instance, the PCA analyses of Figure 4 and Figure S11, Supporting Information, show the potential of quantifying the gas concentration with discrimination and the analysis of gas composition under defined environment, respectively. Therefore, a simple array using three *x*Rh‐TiO_2_/SnO_2_ sensors can be used to estimate the total health impact from aromatic gases.

Third, the sensor array with different catalytic overlayers provides additional benefits for the rational design of artificial olfaction. Various oxide chemiresistors are often used for electronic noses and their optimal sensing temperatures are significantly different from each other. The variation in the sensing temperature can make the design of the sensor array complex and may induce thermal interference between the sensors.^[^ ^42^ ^]^ Furthermore, the loading of catalysts on the entire sensing film often increases the sensor resistance up to an unmeasurable level, hampering cost‐effective circuit design.^[^ ^43^ ^]^ In this study, the same sensing films with different catalytic overlayers were used. Because the sensing reaction and catalytic reaction are well separated, the sensor resistance remains similar and the gas selectivity can be tailored at a constant temperature by simply controlling the overlayer catalysts. Gas sensing patterns can also be modulated by controlling the thickness of the same catalytic overlayer (e.g., 0.5Rh‐TiO_2_, thickness: 6.3 µm) as demonstrated in Figure S12, Supporting Information, which is supported again by the results in our previous works.^[^ ^25^, ^26^, ^27^, ^28^ ^]^ Furthermore, not only thick films but also thin films with catalytic overlayer can be used to control gas selectivity or to establish electronic noses.^[^ ^44^, ^45^ ^]^ Thus, the as‐prepared bilayer sensors provide distinctive advantages for gas sensors and electronic noses. Furthermore, the discrimination and analysis of aromatic gases can be used in a wide range of applications such as gas leak detectors, estimation of BTX exposure in petroleum industries,^[^ ^46^ ^]^ air quality monitoring at gas stations,^[^ ^47^ ^]^ and air pollution assessment in congested areas.^[^ ^48^ ^]^

To understand the gas‐sensing mechanism, the contribution of Rh‐TiO_2_ catalysts in the reforming and oxidation of aromatic compounds was investigated using PTR‐QMS. For this, 0.01 g of *x*Rh‐TiO_2_ powder was loaded on quartz wool placed in the middle of a quartz tube and kept within a furnace. The conversion (*η*) of 1 ppm benzene, toluene, or *p*‐xylene in the absence and presence of *x*Rh‐TiO_2_ catalysts was calculated at various temperatures using the equation *η* = (1 ‐ [C]_out_ / [C]_in_) × 100(%), where [C]_in_ and [C]_out_ are the concentrations of gas before and after reaction at high temperature, respectively.

**Figure 5:**
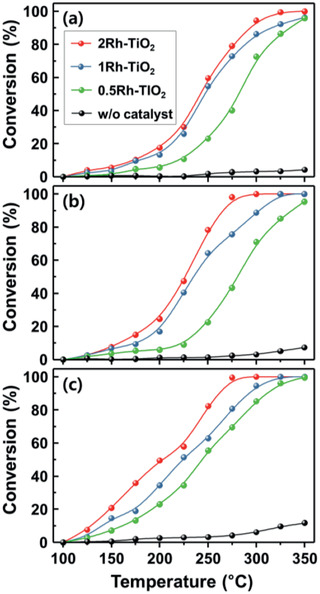
Catalytic performance of *x*Rh‐TiO_2_ (2Rh‐TiO_2_; red line, 1Rh‐TiO_2_; blue line, 0.5Rh‐TiO_2_; green line, without catalyst; black line) catalysts in the conversion of 1 ppm a) benzene, b) toluene, and c) *p*‐xylene in the range 100–350 °C.

The conversion amounts of benzene, toluene, and *p*‐xylene without the catalysts were low (4.3%, 7.3%, and 11.7%) at 350 °C (**Figure** **5**). In contrast, the Rh‐TiO_2_ catalysts drastically promoted the conversion of aromatic compounds, and with increasing Rh loading on the TiO_2_ support, the amount of conversion increased. For example, the temperature for the 50% and 90% conversion (*T_*η*_*_,gas__=50%_ and *T_*η*_*_,gas__=90%_) of benzene, toluene, and *p*‐xylene decreased with increasing the loading of Rh from 0.5 to 2 wt% (Figure 5, and Table S2, Supporting Information), verifying the catalytic role of Rh. In addition, the order of temperatures for the 50% and 90% conversions (*T_*η*_*_,B__=50%_ ≥ *T_*η*_*_,T__=50%_ ≥ *T_*η*_*_,X__=50%_, *T_*η*_*_,B__=90%_ ≥*T_*η*_*_,T__=90%_≥ *T_*η*_*_,X__=90%_) indicates the reactivity of the aromatic compounds with Rh‐TiO_2_ (X ≥ T ≥ B).

The by‐products of the reaction in the absence and presence of the 0.5Rh‐TiO_2_ catalyst were analyzed using PTR‐QMS. Aromatic compounds are known to produce various intermediate species during catalytic reaction at elevated temperatures, including alcohols, aldehydes, acetates, and other aromatics.^[^ ^49^, ^50^, ^51^, ^52^ ^]^ In this study, many different intermediate phases were also observed but the main by‐products at the reactor outlet, whose ion signals increased with decreasing aromatic compound concentrations, were acetaldehyde, toluene, C9 aromatics, C10 aromatics, benzene, and benzaldehydes (Figure S13, Supporting Information).

**Figure 6:**
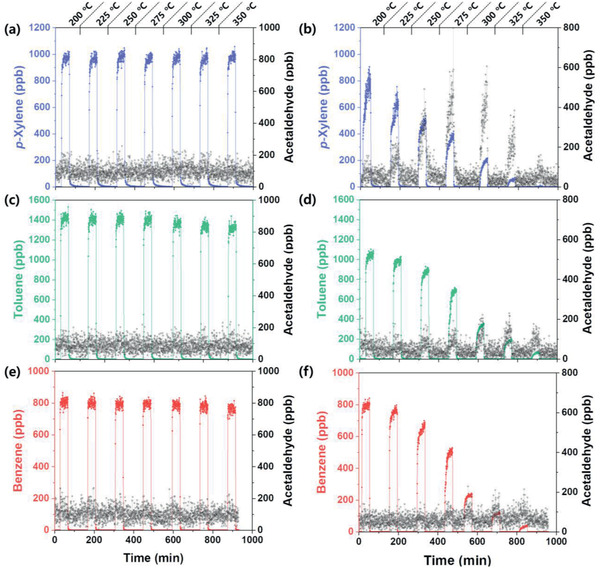
Outlet gas concentrations of aromatic compounds and acetaldehyde in the absence of catalysts measured by PTR‐QMS: a) *p*‐xylene, c) toluene, and e) benzene, and in the presence of catalysts: b) *p*‐xylene, d) toluene, and f) benzene (concentration of inlet gas: 1 ppm, temperature range: 200–350 °C).

Among these, acetaldehyde was detected in all three samples. Considering the high reactivity in the gas‐sensing reaction,^[^ ^53^, ^54^, ^55^ ^]^ acetaldehyde can be regarded as a plausible by‐product, which enhances the gas response of aromatic compounds, although further research is required to elucidate the detailed mechanism of the same and to understand the differences of gas reforming environments in bilayer sensor and quartz tube reactor. **Figure** **6** shows the outlet concentrations of aromatic compounds (*p*‐xylene, toluene, and benzene) and acetaldehyde in the range 200–350 °C. In the absence of a catalyst, the *p*‐xylene and acetaldehyde concentrations in the outlet gas did not change as the reactor temperature increased from 200 to 350 °C (Figure 6a). In contrast, in the presence of a 0.5Rh‐TiO_2_ catalyst, with increasing temperature from 200 to 275 °C, the *p*‐xylene concentration decreased while the acetaldehyde concentration increased (Figure 6b). This clearly suggests that *p*‐xylene was reformed into the more reactive acetaldehyde by a catalytic reaction. When the temperature was increased above 300 °C, the acetaldehyde concentration at the outlet decreased, possibly due to its complete oxidation. This strongly supports the enhancement in the gas response at moderate temperature by gas reforming and the decrease in the gas response at high temperature by complete oxidation. Toluene and benzene showed similar behaviors, but the minimum temperature required to generate acetaldehyde was in the order X < T < B, and the amount of acetaldehyde generated was in the order X > T > B (Figure 6c–f). Relatively low concentrations of outlet acetaldehyde in Figure 6d,f despite the decrease of stock gas concentrations with increasing temperature can be attributed to the less reforming of toluene (or benzene) with low reactivity into acetaldehyde. Above results again support the hypothesis that the formation of acetaldehyde and its concentration are associated with the reactivity of the source gas.

**Figure 7:**
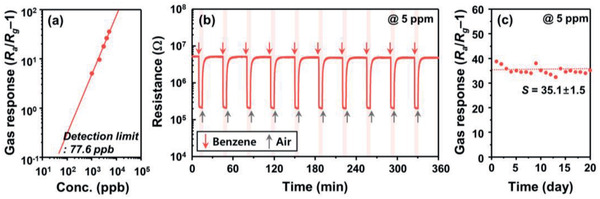
a) Gas response as a function of benzene concentration (1–5 ppm), b) repeated sensing transient to 5 ppm benzene at 325 °C, and c) long‐term stability of the 2Rh‐TiO_2_/SnO_2_ sensor.

**Figure 8:**
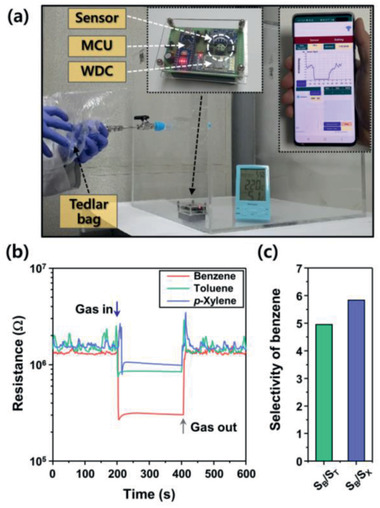
a) Simulated condition for indoor air monitoring (relative humidity: 51% at 22 °C) and wireless sensor module used to detect aromatic compounds (benzene, toluene, and *p*‐xylene), b) dynamic sensing transients, and c) selectivity of benzene over toluene and *p*‐xylene by the sensing module wirelessly connected to a smartphone (MCU: micro‐controller unit; WDC: wireless data communication).

To evaluate the potential for practical applications, the detection limit and stability of the 2Rh‐TiO_2_/SnO_2_ sensor were investigated. The 2Rh‐TiO_2_/SnO_2_ sensor showed a high response of 5.0 to 1 ppm of benzene (**Figure** **7**a). From the gas response to 1–5 ppm of benzene at 325 °C, the detection limit was calculated to be 77.6 ppb when *S* > 0.2 was used as the criterion for gas‐sensing. The sensor showed reproducible sensing transients upon repeated exposure to 5 ppm of benzene and exhibited long‐term stability over 20 days (Figure 7b,c). The gas‐sensing characteristics under simulated indoor conditions (static atmosphere, relative humidity (RH) 51%, temperature 22 °C) were also investigated. For this, a portable sensing module that is wirelessly connected to a mobile phone was placed in a chamber (inner volume: 50 × 50 × 50 cm^3^), and the ambient humidity was controlled by a humidifier (**Figure** **8**a). When the sensor resistance is stabilized, a particular volume of benzene, toluene, or *p*‐xylene (100 ppm/air balance, 6.25 L, collected by Tedlar bags) was injected into the chamber, and the sensor resistance was measured. After dilution, the final concentrations of benzene, toluene, and *p*‐xylene were fixed at 5 ppm. The 2Rh‐TiO_2_/SnO_2_ sensor exhibited high selectivity toward benzene over other aromatic compounds (*S*_benzene_/*S*_toluene_ = 4.95, and *S*_benzene_/*S_p_*_‐xylene_ = 5.83) under the simulated conditions (Figure 8b,c). In order to assess the gas sensing characteristics in real environment, the sensing transients upon 5 ppm of benzene under different humidity conditions (RH 41%, RH 51%, and RH 80% at 22 °C) were measured. Although the sensor showed the decrease of gas response with increasing humidity (Figure S14, Supporting Information), the gas response remained substantial even in highly humid atmosphere (RH 80%). These results demonstrate that the fabricated sensor can be used to detect sub‐ppm‐level benzene under a varied‐humidity atmosphere.

## 3 Conclusion

Tailored control over the response and selectivity to aromatic BTX compounds has been achieved using bilayer sensors consisting of a SnO_2_ sensing layer and Rh‐TiO_2_ composite catalytic overlayers. The SnO_2_ sensor coated with 2 wt% Rh‐TiO_2_ enabled highly selective detection of ppm‐ and sub‐ppm‐levels of carcinogenic benzene with negligible cross‐response to other indoor pollutants, such as toluene, *p*‐xylene, HCHO, CO, and ethanol. Furthermore, the array containing three sensors with 0.5, 1, and 2 wt% Rh‐TiO_2_ overlayers demonstrated systematic control over the response and selectivity toward aromatic compounds, enabling the rational design of an electronic nose for the discrimination of benzene, toluene, and *p*‐xylene and the composition analysis of mixed aromatic gases in a defined environment. Based on the PTR‐MS analysis, the enhancement and suppression of gas response by the reforming and oxidation of analyte gases, respectively, obtained by the tailored catalytic activation of the Rh‐TiO_2_ overlayer, could lead to the on‐demand design of ultra‐selective benzene sensors and high‐performance electronic noses to assess the total health impacts of aromatic gases. For the design of highly robust gas sensors and electronic noses, the endurance of sensor against ambient moisture should be improved further. The sensors and sensor array fabricated in this study are expected to open up a new avenue for accurate indoor air quality monitoring, gas leak detection in the petroleum industry, and assessment of air pollution in congested areas and gas stations.

## 4 Experimental Section

##### Materials

SnO_2_ hollow spheres were prepared by ultrasonic spray pyrolysis. A sample containing 0.1 mol of SnCl_2_·2H_2_O, (≥99.995%, Sigma‐Aldrich, U.S.A) and 0.25 mol of C_6_H_8_O_7_·*x*H_2_O (≥ 99.0%, Sigma‐Aldrich, U.S.A) was dissolved in 500 mL of HCl solution (35.0–37.0%, HCl:H_2_O = 1:99 by vol%, Sigma‐Aldrich, U.S.A). Droplets of the sprayed solution nebulized by six ultrasonic transducers were transported to a quartz reactor (temperature: 700 °C). As‐prepared precursor spheres were converted to SnO_2_ hollow spheres by heat treatment at 600 °C for 2 h in air. TiO_2_ powder loaded with different amounts of Rh ([Rh]/[Sn] = 0.5, 1, and 2 wt%) was prepared by the impregnation method. Next, 0.1 g of TiO_2_ (P25, average particle size: 21 nm, Degussa, Germany) was mixed with 100 mL of distilled water, and then 0.5–2 wt% RhCl_3_·*x*H_2_O (99.98%, Sigma‐Aldrich, U.S.A) was added. The mixture was stirred at room temperature for 6 h and dried at 80 °C overnight. The powder was annealed at 500 °C for 2 h in air. The as‐prepared SnO_2_ powders were mixed with terpineol‐based ink vehicle (FCM, U.S.A) using a mortar and pestle. The SnO_2_ sensing film was screen‐printed onto alumina substrates (thickness: 0.25 mm, area: 1.5 mm × 1.5 mm) with two gold electrodes (electrode gap: 0.2 mm) on the top surface and a microheater on the bottom. After screen printing, the green film was dried at 200 °C for 2 h. The Rh‐loaded TiO_2_ slurry was prepared in the same way and screen‐printed onto the surface of the SnO_2_ films. The films were annealed at 500 °C to obtain a bilayer sensor. For simplicity, the SnO_2_ sensor with the *x*Rh‐loaded TiO_2_ overlayer was represented as xRh‐TiO_2_/SnO_2_ (*x* = 0.5–2 wt%).

##### Catalytic Evaluation

A schematic diagram of the experimental apparatus used for catalytic evaluation is shown in Figure S15, Supporting Information. The catalytic activities of the *x*Rh‐TiO_2_ powder were evaluated in a quartz tube reactor (length: 400 mm, inner diameter: 8 mm) kept within a furnace. 0.01 g of *x*Rh‐TiO_2_ powder was loaded onto the quartz wool bed placed in the middle of the quartz tube. The total flow rate of the reactant gases (1000 ppb benzene, toluene, and *p*‐xylene, 21% O_2_/N_2_) was 200 cm^3^ min^−1^. The reactant and by‐product gases were analyzed by on‐line PTR‐QMS (PTR‐QMS 300, Ionicon Analytik, Austria). The drift tube conditions were fixed (voltage: 600 V, temperature: 80 °C, pressure: 2.3 mbar) and the electric field strength/gas number density (*E*/*N*) was 136 Td (1 Td = 10^−17^ V cm^2^). The H_3_O^+^ ions served as a primary ion, and the temperatures for catalytic activity testing ranged from 100 to 350 °C. The procedures for data acquisition and analysis of sensor data, and materials characterization are shown in the Experimental Section, Supporting Information.

## Conflict of Interest

The authors declare no conflict of interest.

## Supporting information

Supporting Information
