# Supplementary material for: Highly Selective Detection of Benzene and Discrimination of Volatile Aromatic Compounds Using Oxide Chemiresistors with Tunable Rh‐TiO2 Catalytic Overlayers
Source: Adv Sci (Weinh). 2021 Feb 1;8(6):2004078. doi: 10.1002/advs.202004078 (PMC7967053; doi:10.1002/advs.202004078)
Supplement: Supplementary file 1 — Supporting Information [file ADVS-8-2004078-s001.pdf]

© 2021 Wiley-VCH GmbH

ADVANCED SCIENCE
Open Access

Supporting Information

for *Adv. Sci.,* DOI: 10.1002/advs.202004078

Highly Selective Detection of Benzene and Discrimination of Volatile Aromatic Compounds Using Oxide Chemiresistors with Tunable Rh-TiO2 Catalytic Overlayers

*Young Kook Moon, Seong-Yong Jeong, Young-Moo Jo, Yong Kun Jo, Yun Chan Kang, and Jong-Heun Lee**

© Copyright 2021. WILEY-VCH GmbH.

Supporting Information

Highly Selective Detection of Benzene and Discrimination of Volatile Aromatic Compounds Using Oxide Chemiresistors with Tunable Rh-TiO_2 Catalytic Overlayers

*Young Kook Moon, Seong-Yong Jeong, Young-Moo Jo, Yong Kun Jo, Yun Chan Kang, and Jong-Heun Lee**

Department of Materials Science and Engineering, Korea University, Seoul 02841, Republic of Korea
*E-mail: jongheun@korea.ac.kr

Experimental Section

Sensor Data Acquisition and Analysis of Sensor Data: The sensors were heat-treated at 500 °C for 2 h for thermal stabilization prior to measurement. They were placed in a specially designed quartz tube (inner volume: 1.5 cm^3). Gas concentrations were controlled by mixing the analyte gas and synthetic air using an automatic gas mixing system, and the gas atmosphere was switched by a four-way valve to ensure a constant flow rate (200 cm^3 min^{-1}). The DC two-probe resistance was measured using an electrometer (6487 picoammeter/voltage source, Keithley, USA) interfaced with a computer. The gas responses (R_a/R_g–1, R_a: resistance in air, R_g: resistance in gas) to 5 ppm of benzene, toluene, *p*-xylene, ethanol, formaldehyde, and carbon monoxide were measured in the range 325–425 °C. The principal components analysis (PCA) was used for pattern recognition to classify multivariant sensor data. PCA is a method of finding the axis that best describes all variables by obtaining the covariance matrix and performing eigenvalue decomposition. A data matrix for single analyte was constructed by gas response data from three sensors (0.5Rh-TiO_2/SnO_2, 1Rh-TiO_2/SnO_2, and 2Rh-TiO_2/SnO_2) for 1–5 ppm of benzene, toluene, *p*-xylene, ethanol, HCHO, and CO at 325 °C. From this matrix, the eigenvalue and eigenvector were calculated. Each observation was projected onto this eigenvector line to get a coordinate value along the principal component line. The first principal component (PC1) represents the maximum variance direction in the data. A second PC (PC2) exhibits the second-largest variance along the direction orthogonal to PC1. After calculating the third PC (PC3: orthogonal to both PC1 and PC2), the percentages of PC1, PC2, and PC3 out of the total component (PC1+PC2+PC3) were calculated. Since the sum between PC1 and PC2 was sufficiently high (> 90%), PC1 and PC2 were plotted in Figure 4 and Figure S11. 18 different binary mixtures of aromatic compounds (B+T, B+X, and T+A) were made according to the composition given in Figure S11a and their gas sensing characteristics were measured. Subsequently, the principal components (PC1 and PC2) of sensing data were obtained. The gas sensing characteristics under simulated indoor conditions were obtained by the analog-to-

digital converting of sensing signals using a micro-controller unit (MCU, ATmega328, Microchip Technology Inc., USA) and signal transmission to a smartphone via Bluetooth communication.

Materials Characterization: High-resolution scanning electron microscopy (HR-SEM, SU-70, Hitachi Co. Ltd., Japan) was used to observe the morphologies and microstructures of the materials and sensor films. High-angle annular dark-field-scanning transmission electron microscopy (HAADF–STEM) images and elemental mapping images of the SnO_2 and xRh-TiO_2 powders were obtained using Cs-scanning transmission electron microscopy (Cs-STEM, JEM-ARM200F, JEOL Co. Ltd., Japan). The pore size distribution and specific surface area were measured based on the BET analysis of nitrogen adsorption isotherms (TriStar 3000, Micromeritics, USA). The phase and crystallinity of all the materials were analyzed by XRD (D/MAX-2500 V/PC, Rigaku, Japan) with a Cu Kα radiation source ($\lambda = 1.5418$Å). The chemical states of the TiO_2 loaded with Rh nanoparticles were analyzed using XPS (X-tool, Ulvac PHI, Japan). The cross-sectional images and composition of the 2Rh-TiO_2/SnO_2 sensor were investigated using a field-emission electron probe microanalysis (FE-EPMA, JXA-8530F, JEOL Co. Ltd., Japan) apparatus.

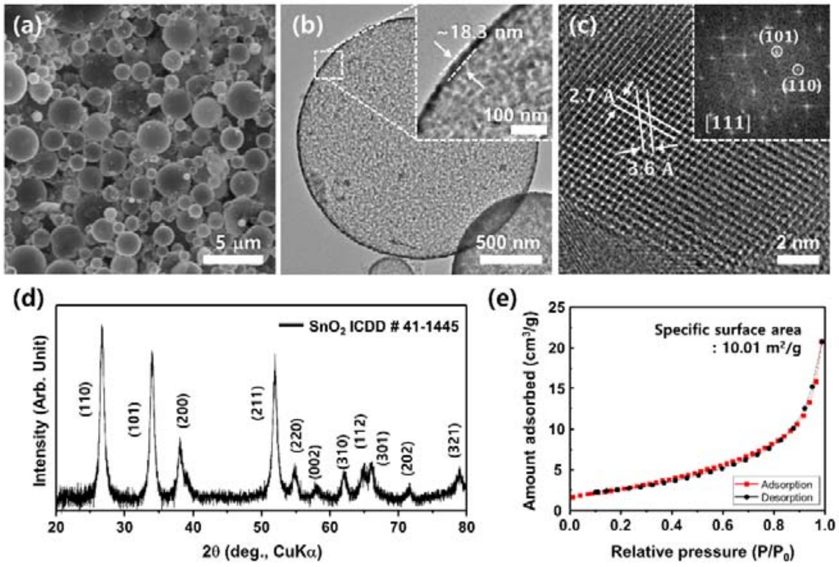

Figure S1. Microstructure and characterization of SnO_2 hollow spheres: a) SEM image, b,c) TEM images, d) XRD pattern, and e) N_2 adsorption/desorption isotherm and specific surface area.

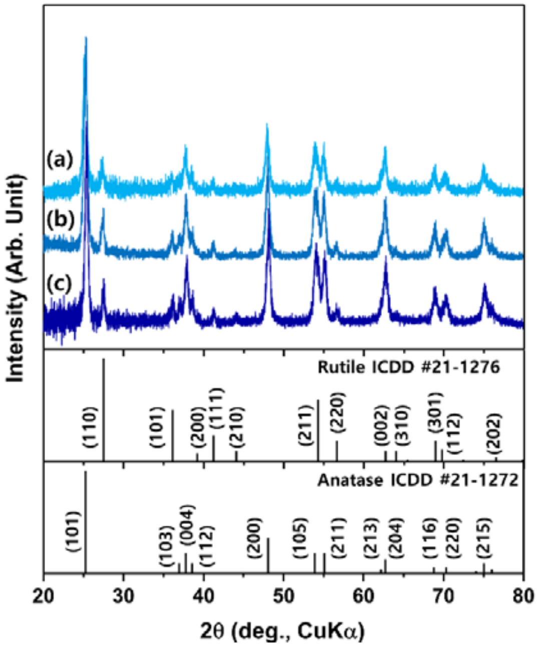

Figure S2. XRD patterns of xRh-TiO_2 (x = a) 0.5, b) 1, and c) 2 wt%) powder.

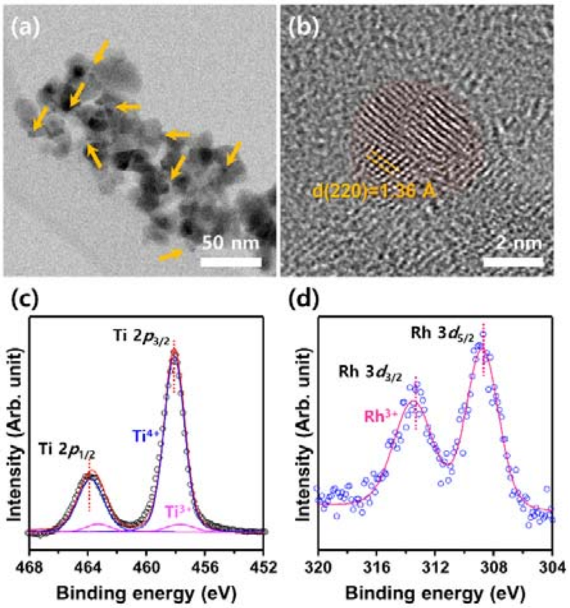

Figure S3. a,b) HRTEM images of the 2Rh-TiO_2 specimen, and XPS profiles of c) Ti 2p and d) Rh 3d of the 2Rh-TiO_2 specimen.

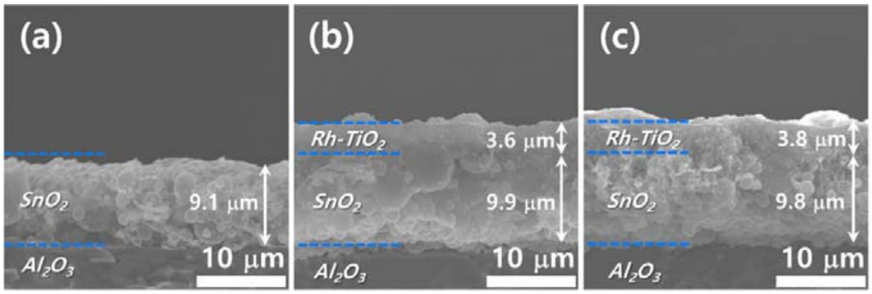

Figure S4. Cross-sectional SEM images of a) SnO_2, b) 0.5Rh-TiO_2/SnO_2, and c) 1Rh-TiO_2/SnO_2 sensors.

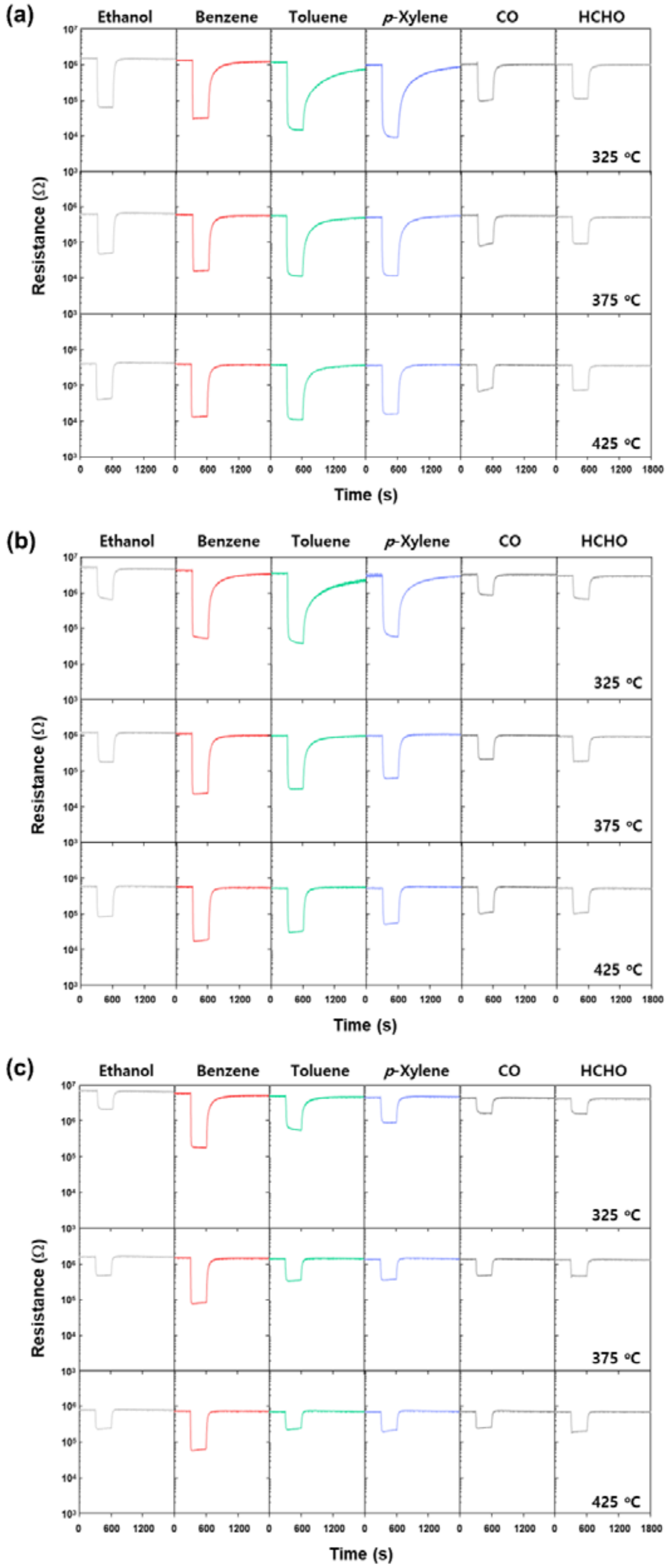

Figure S5. Dynamic sensing transients of a) 0.5Rh-TiO_2/SnO_2, b) 1Rh-TiO_2/SnO_2, and c) 2Rh-TiO_2/SnO_2 sensors to 5 ppm analyte gases.

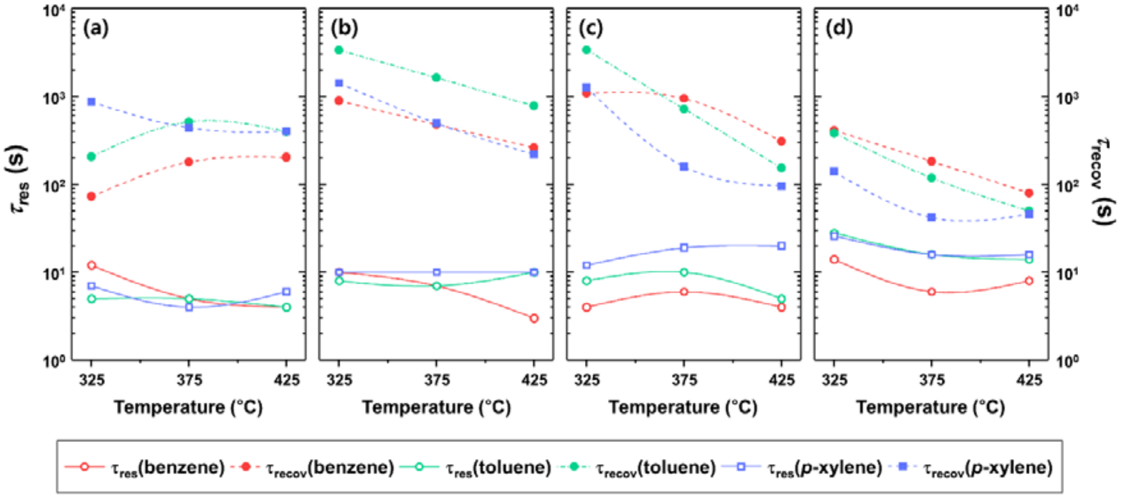

Figure S6. 90% response time (τ_{res}) and 90% recovery time (τ_{recov}) of the a) SnO_2, b) 0.5Rh-TiO_2/SnO_2, c) 1Rh-TiO_2/SnO_2, and d) 2Rh-TiO_2/SnO_2 sensors at 325–425 °C.

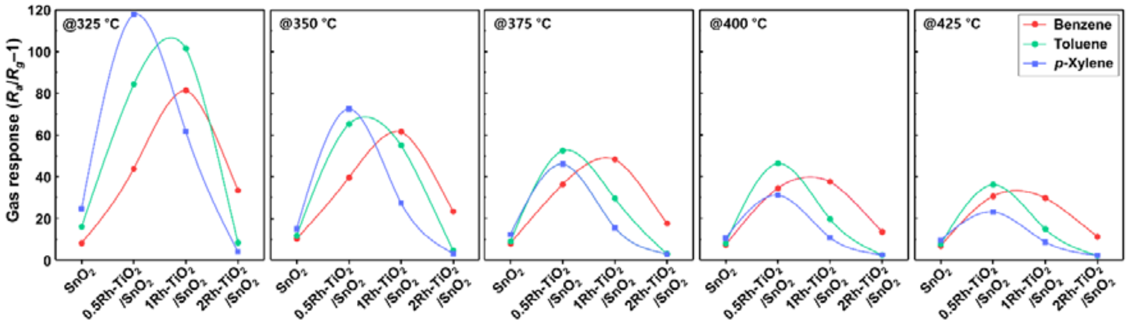

Figure S7. Gas responses to aromatic compounds (concentration: 5 ppm) as a function of the catalyst loading amount (SnO_2, 0.5Rh-TiO_2/SnO_2, 1Rh-TiO_2/SnO_2, and 2Rh-TiO_2/SnO_2 sensors) at 325–425 °C.

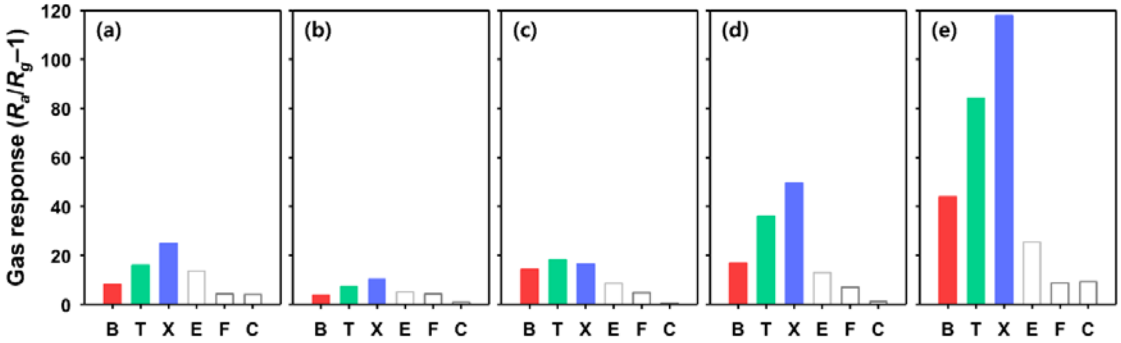

Figure S8. Gas sensing characteristics of a) SnO_2, b) 0.5Pd-TiO_2/SnO_2, c) 0.5Pt-TiO_2/SnO_2, d) 0.5Au-TiO_2/SnO_2, and e) 0.5Rh-TiO_2/SnO_2 sensors to 5 ppm of various gases (benzene (B), toluene (T), *p*-xylene (X), ethanol (E), HCHO (F), and CO(C)) at 325 °C.

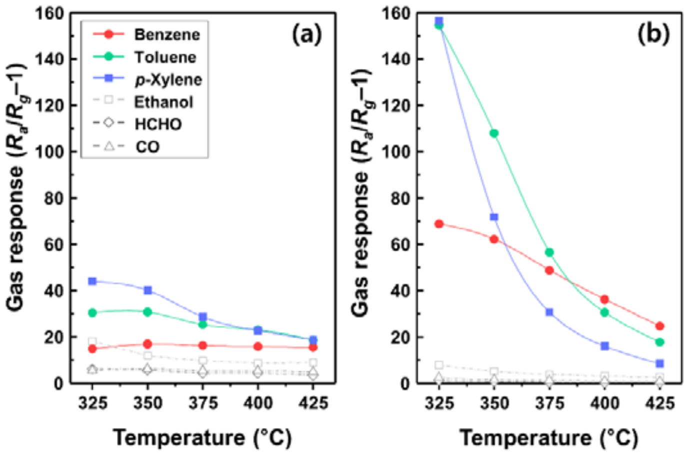

Figure S9. Gas sensing characteristics of a) TiO_2/SnO_2, and b) 2Rh-Al_2O_3/SnO_2 sensors to 5 ppm benzene, toluene, *p*-xylene, ethanol, HCHO, and CO in the range 325−425 °C.

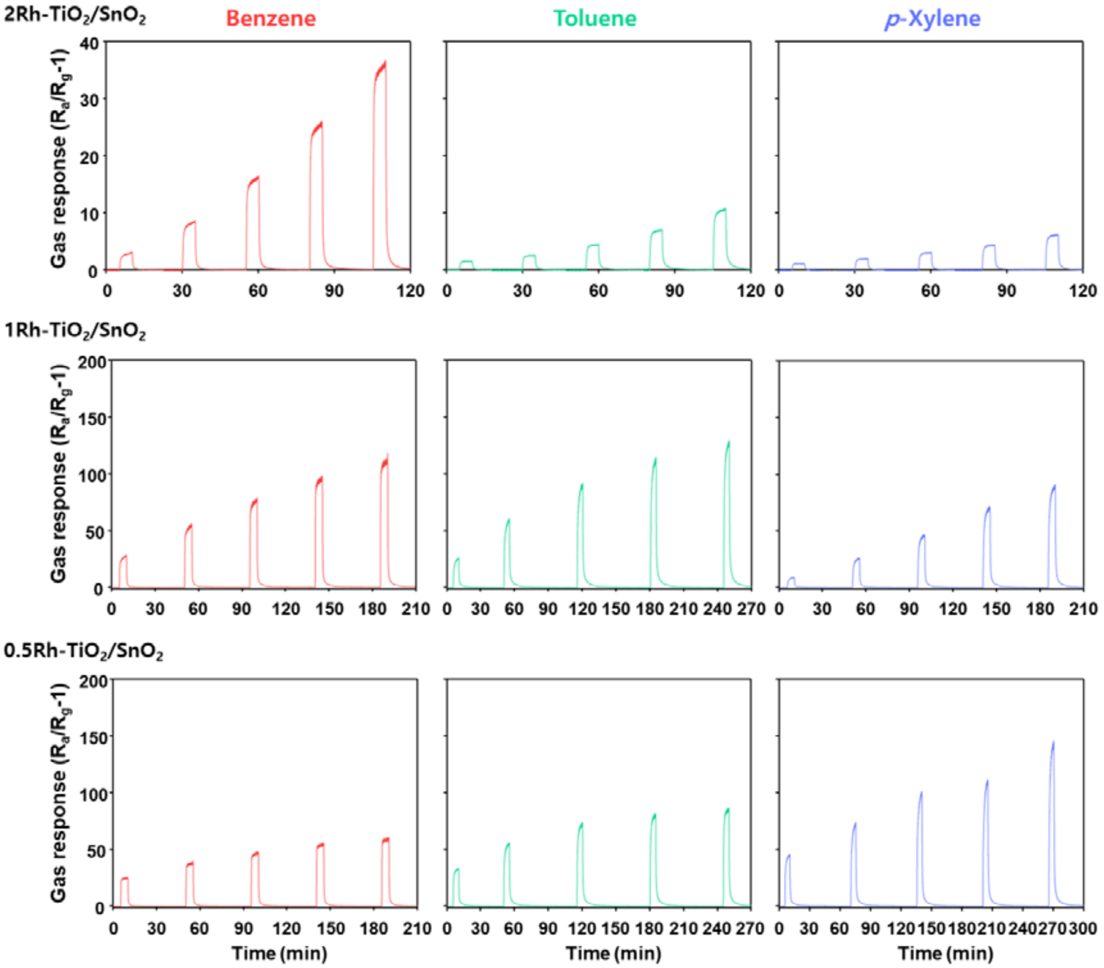

Figure S10. Representative sensing traces showing the response of 0.5−2 wt% Rh-TiO_2/SnO_2 sensors to 1−5 ppm of benzene, toluene, and *p*-xylene at 325 °C.

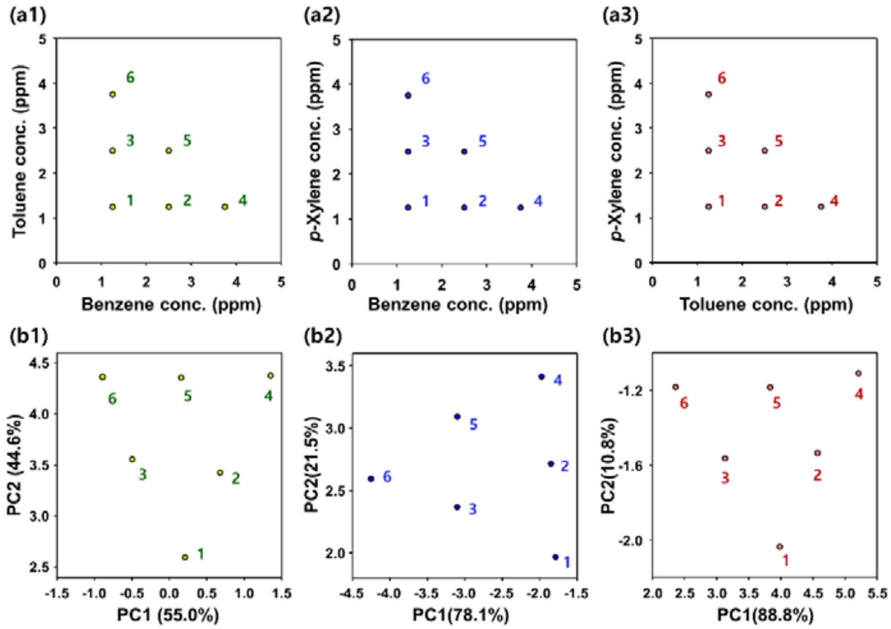

Figure S11. Detection of binary mixtures of benzene, toluene, and *p*-xylene. Experimental plans (gas composition for test) of a1) benzene + toluene, a2) benzene + *p*-xylene, and a3) toluene + *p*-xylene, and PCA plots using the gas sensing data of b1) benzene + toluene, b2) benzene + *p*-xylene, and b3) toluene + *p*-xylene.

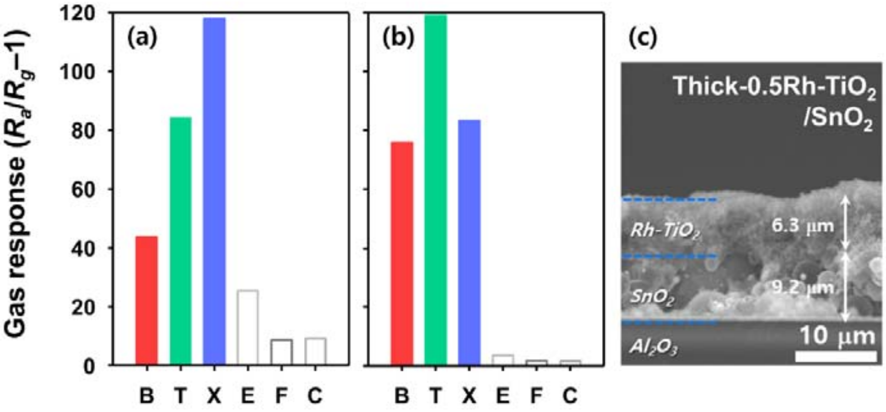

Figure S12. Gas sensing characteristics of a) 0.5Rh-TiO_2/SnO_2 and b) thick-0.5Rh-TiO_2/SnO_2 sensors to benzene (B), toluene (T), *p*-xylene (X), ethanol (E), HCHO (F), and CO(C) at 325 °C. c) Cross sectional SEM image of thick-0.5Rh-TiO_2/SnO_2 sensor.

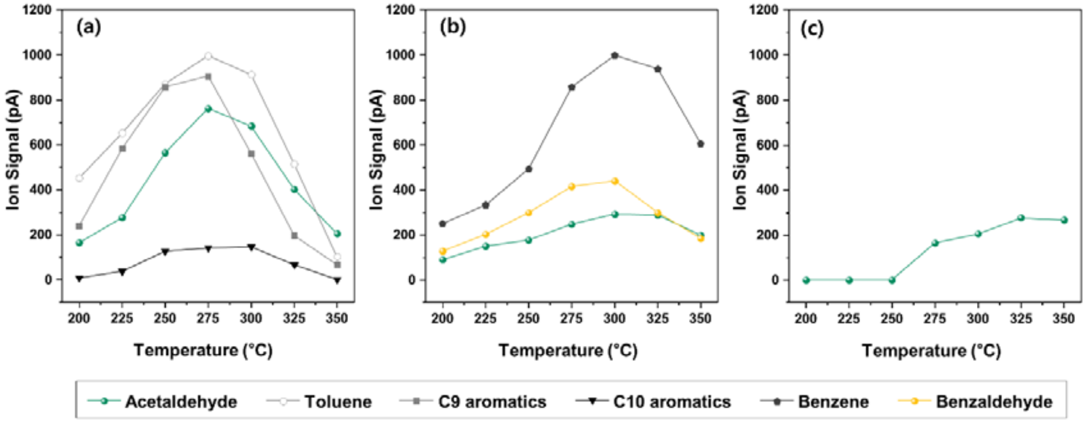

Figure S13. Main by-product species identified and quantified at the gas outlet by PTR-QMS (inlet gas: 1 ppm a) *p*-xylene, b) toluene, and c) benzene). (catalyst: 0.5Rh-TiO_2)

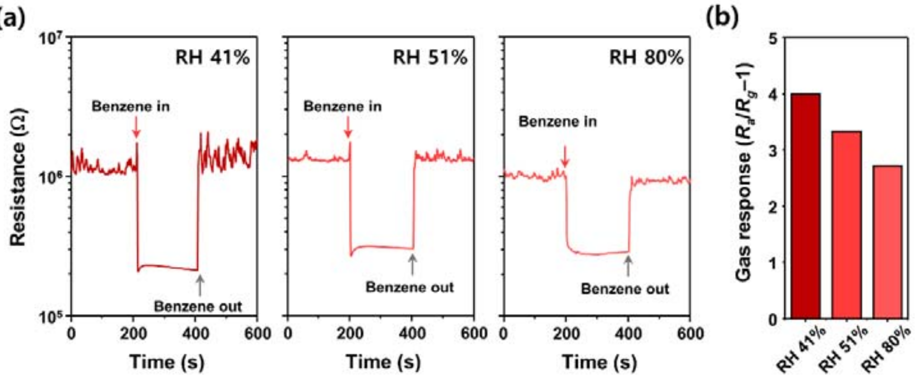

Figure S14. a) Gas sensing transients and b) gas response of the 2Rh-TiO_2/SnO_2 sensor to 5 ppm of benzene under different humidity conditions (relative humidity: 41%, 51%, and 80% at 22°C). An acrylic chamber with a fixed volume (inner volume: 50 × 50 × 50 cm^3) was used for measuring the gas sensing characteristics. The humidity in the chamber was controlled using a humidifier.

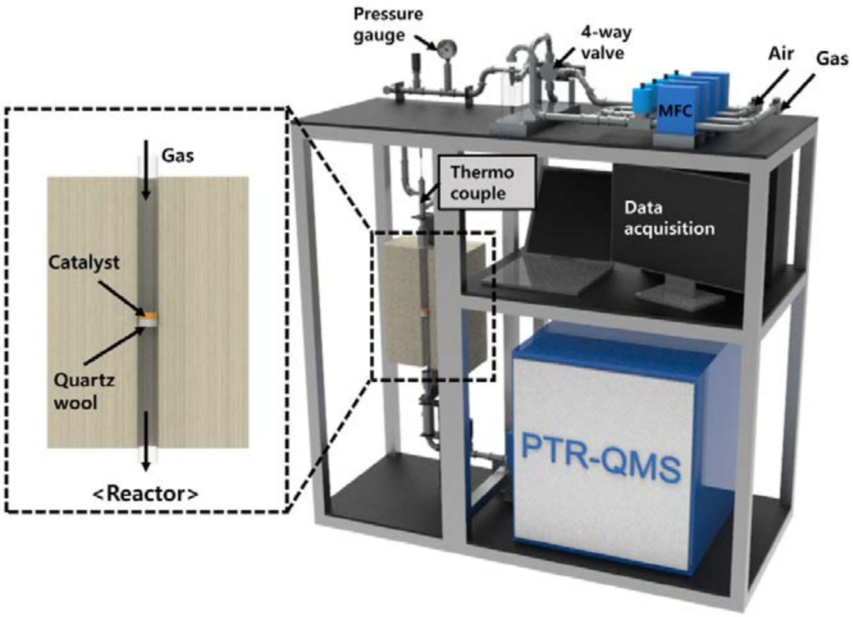

Figure S15. Schematic diagram of the PTR-QMS equipment setup.

Table S1. BET specific surface area of the prepared samples (0.5Rh-TiO_2, 1Rh-TiO_2, 2Rh-TiO_2, and 2Rh-Al_2O_3)

Catalysts	BET specific surface area [m^2 g^{-1}]
0.5Rh-TiO_2	28.17
1Rh-TiO_2	32.02
2Rh-TiO_2	22.94
2Rh-Al_2O_3	84.39

Table S2. Catalytic performance test results of the prepared samples (2Rh-TiO_2, 1Rh-TiO_2, and 0.5Rh-TiO_2). Temperature at which aromatic compounds undergo 50% conversion ($T_{\eta,gas=50\%}$) and 90% conversion ($T_{\eta,gas=90\%}$).

Catalysts	Aromatic compounds	$T_{\eta,gas=50\%}$ [°C]	$T_{\eta,gas=90\%}$ [°C]
2Rh-TiO_2	Benzene	241	294
	Toluene	227	266
	p-Xylene	203	258
1Rh-TiO_2	Benzene	247	317
	Toluene	236	304
	p-Xylene	223	290
0.5Rh-TiO_2	Benzene	281	336
	Toluene	281	336
	p-Xylene	243	308
